# Serum progesterone levels greater than 32.5 ng/ml on the day of embryo transfer are associated with lower live birth rate after artificial endometrial preparation: a prospective study

**DOI:** 10.1186/s12958-021-00703-6

**Published:** 2021-02-18

**Authors:** Ashraf Alyasin, Marzieh Agha-Hosseini, Motahareh Kabirinasab, Hojatollah Saeidi, Maryam Shabani Nashtaei

**Affiliations:** 1 Department of Infertility, Shariati Hospital, Tehran University of Medical Sciences, Tehran, Iran; 2 Department of Obstetrics and Gynecology, Shariati Hospital, Tehran University of Medical Sciences, 1411713135, Shariati Hospital, Jalal-e-Al-e-Ahmad Hwy, Tehran, Iran; 3 Department of Biology and Embryology, Omid Fertility Center, Tehran, Iran; 4 Department of Anatomy, School of Medicine, Tehran University of Medical Sciences, Tehran, Iran

**Keywords:** Serum progesterone, Estradiol, Frozen‐warmed embryo transfer, Live birth rate

## Abstract

**Background:**

Previous observational studies have highlighted the negative effects of serum hormone levels at the minimum threshold during frozen embryo transfer (FET) cycles. However, still the questions regarding the maximum threshold level, and the highest allowed dosage of hormonal medications remain unresolved. The present study was conducted to determine whether there is any relationship between the serum progesterone and estradiol levels on the day of ET, and live birth rate (LBR) in patients receiving HRT in FET cycles.

**Methods:**

In this prospective cohort study, eligible women who were undergoing their first or second FET cycles with the top graded blastocyst stage embryos were included. All patients received the same HRT regimen. FET was scheduled 5 days after administration of the first dosage of progesterone. On the morning of ET, 4–6 h after the last dose of progesterone supplementation, the serum progesterone (P_4,_ ng/ml) and estradiol (E_2_, pg/ml) levels were measured.

**Results:**

Amongst the 258 eligible women that were evaluated, the overall LBR was 34.1 % (88/258). The serum P_4_ and E_2_ values were divided into four quartiles. The means of women’s age and BMI were similar between the four quartiles groups. Regarding both P_4_ and E_2_ values, it was found that the LBR was significantly lower in the highest quartile group (Q_4_) compared with the others, (*P* = 0.002 and *P* = 0.042, respectively). The analysis of the multivariable logistic regression showed that the serum level of P_4_ on ET day, was the only significant predictive variable for LBR. The ROC curve revealed a significant predictive value of serum P_4_ levels on the day of ET for LBR, with an AUC = 0.61 (95 % CI: 0.54–0.68, *P* = 0.002). The optimum level of serum P_4_, with 70 % sensitivity and 50 %specificity for LBR, was 32.5 ng/ml.

**Conclusions:**

The present study suggests that a serum P4 value at the maximum threshold on the day of FET is associated with reduced LBR following blastocyst transfer. Therefore, measuring and monitoring of P_4_ levels during FET cycles might be necessary. However, the results regarding the necessity for the screening of serum E_2_ levels before ET, are still controversial, and further prospective studies are required.

## Introduction

Recently, the “freeze –all” strategy has been employed by most fertility clinics due to the recent advances in laboratory procedures for cryopreservation and thawing of embryos and reducing the risk of ovarian hyper-stimulation syndrome. After the optimization of embryos’ quality and survival rate, the endometrial preparation is important. Although, the advantages of any particular endometrial preparation protocol for frozen embryo transfer (FET) over others has not been determined as yet, many clinicians prefer to use hormonal replacement therapy (HRT) for artificial endometrial preparation as it needs [1] less control monitoring, allows for flexibility of scheduling, has a lower cycle cancellation rate and is cost-effective [2]. However, a proper synchronization between the embryo and the endometrium is still required by controlling the timing and the dosage of exposure to exogenous progesterone [2].

Recently, the link between the level of serum progesterone and estradiol in luteal phase and the pregnancy outcome in HRT-FET cycles has been explored. A retrospective study conducted by Yovich et al., suggested an optimal window of progesterone level (from 70 to 99 nmol/l) is associated with the highest pregnancy rate [3]. More recently, a few studies have been conducted to find out whether there is any optimal level of progesterone around the ET day [2, 4–8] and on the pregnancy test day [9]. There is limited data regarding the administration of vaginal progesterone, however; a similar debate exists regarding the intramuscular (IM) route [6]. The current data suggests both low [2, 4, 6, 7, 10] and high [5] levels of serum progesterone on ET day result in a lower pregnancy rate, indicating different cut-off points in progesterone levels on ET day can predict the outcome of pregnancy. In most of the studies, patients receive either vaginal administration of P [2, 3, 6, 7, 10] or an IM administration [4–6], but the combined approach of both methods has not been investigated.

Coroleu and Gaggiotti-Marre have speculated that a certain serum P value should be attained for an adequate immunological environment to allow implantation to occur and reduce pregnancy loss in FET cycles [11]; however, Polat et al., in a recent retrospective study, reported that neither the circulating P level nor the type of progesterone administration were independent predictors of ongoing pregnancy rate [12]. Therefore, based on existing research, the relationship between the serum progesterone levels on ET day and pregnancy outcomes as well as a certain dose of progesterone to achieve the most optimal clinical pregnancy and live birth rate are debatable topics.

This prospective study was conducted to determine the nature of the relationship between serum progesterone and estradiol levels on the day of ET and the live birth rate in patients receiving both vaginal and IM progesterone together for HRT in FET cycles.

## Materials and methods

### Methods

This prospective cohort study was carried out in infertile women who underwent frozen embryo transfer in a teaching center affiliated with Tehran University of Medical Sciences (Shariati Hospital) and a private infertility centre (Omid clinic) between February 2019 and February 2020. The study was approved by the Institutional Review Board and Ethics Committee of the Tehran University of Medical Sciences. Eligible women included in this study were aged < 40 years, with a body mass index < 30, and undergoing their first or second frozen embryo transfer cycle using the freeze-all strategy, with 1–2 top quality blastocysts. Patients with oocyte or embryo donation cycles, recurrent miscarriages and implantation failure, severe male factor, uterine diseases or hydrosalpinx diagnosis were excluded from the study. The patients were selected in order of appearance according to their convenient accessibility, and each patient participated in the study only once and on the condition of written consent.

All the patients received hormone replacement therapy for endometrial preparation without pituitary down-regulation with a GnRH agonist. An ultrasound scan and E_2_ measurement was carried out on days 2–3 of the spontaneous menstrual cycle to confirm pituitary desensitization. The endometrial preparation was started using 6 mg estradiol valerate daily if the endometrial thickness was less than 5 mm and serum estradiol level < 50 pg/ml. After 10–12 days of estradiol administration and if ultrasound showed an optimal endometrial thickness (≥ 7 mm, with a triple*-*line pattern), estradiol valerate was continued at the same doage and then a vaginal progesterone suppository (Cyclogest® 400 mg, Actoverco, Iran) was administered once on the first day, twice on the second day, and thrice for the following days. Meanwhile, starting on the fourth day, 25 mg of progesterone (Aburaihan Pharmaceutical Co., Tehran, Iran) was administered intramuscularly (IM), and then was continued at 50 mg per day in the following days. Otherwise, the estradiol dosage was increased to 8 mg/day to achieve the appropriate endometrial thickness. The hormone therapy was continued until a pregnancy test was performed, and in cases of a positive pregnancy, estradiol valerate and progesterone continued to be administered until week 10 of gestation. All sonographic evaluations were performed by an expert infertility fellowship using a Philips Affiniti 70 ultrasound machine with a C10-3v Pure-Wave endo-vaginal probe. According to the women’s age, up to two frozen embryos were thawed and transferred at the blastocyst stage on the 5th day of progesterone treatment. On the day of ET, eligible patients were selected for this study. After checking all the inclusion and exclusion criteria, patients were informed about the objective of the study, and asked to sign a written consent enrollment form.

### Embryo morphology assessment

The vitrification/warming protocol was performed according to the method described previously [13]. All the vitrified blastocysts were thawed on day 5 and transferred on the same day. The grading for blastocysts is based the inner cell mass and trophectoderm appearance assessment, as proposed by Gardner et al. [14]. In the present study only patients who had the top grading (high and medium level) embryos, according to the Gardner classification, were included. The blastocyst embryos were cultured in 10 µl of culture media (Sage Biopharma, Gytech, Australia) under mineral oil at 37 °C and 6 % CO2, 5 % O2, and nitrogen balance in K-System incubator (K-System G210, CooperSurgical, USA). For the transfer, a pre-equilibrated Universal transfer medium (UTM) with phenol red (MediCult) was used.

### The hormonal assessment

On the morning of embryo transfer, 4–6 hours after the last dose of progesterone supplementation, the serum progesterone and estradiol levels were measured. All the laboratory tests were performed in the private infertility center (Omid clinic). Using the VIDAS® Progesterone (PRG) assay, progesterone levels were determined. The VIDAS (VITEK® ImmunoDiagnostic Assay System) instrument is an enzyme-linked immunofluorescence assay (ELIFA). The range of measurement of the VIDAS Progesterone reagent was 0.25–80 ng/ml. Progesterone values below the limit of detection (< 0.25 ng/ml) were read as half the limit of detection (i.e., 0.125 ng/ml), and a control and standardisation were run once daily. Meanwhile, serum levels of estradiol were measured using an automated Elecsys Immunoanalyzer (Roche Diagnostics, Mannheim, Germany). Intra-and inter-assay variation coefficients were less than 5 % and less than 10 %, respectively. All of the hormonal measurement procedures including the preparation setup, dilution and regulation, assay, and quality control processes were carried out according to the manufacturer’s instructions.

### Outcome measures

The primary outcome was determining the relationship between serum progesterone (P_4,_ ng/ml) and estradiol (E_2_, pg/ml) levels on the day of ET and live birth rate (LBR) in artificial endometrial preparation cycles. The secondary outcomes included the clinical pregnancy rate (CPR) (the number of pregnancies with presence of a gestational sac with fetal heartbeat on vaginal ultrasound per ET cycles), blighted ovum rate (the number of pregnancies per ET cycle in which the embryo fails to develop or is reabsorbed), miscarriage rates (the number of the spontaneous loss of a clinical pregnancy before week 20 per ET cycles) and LBR (the number of deliveries that resulted in at least one live born fetus per ET cycle).

### Statistical analysis

Statistical analysis was done using the Statistical Package for Social Sciences (SPSS Inc., Chicago, IL, USA) version 21.0. The serum levels of P_4_ and E_2_ on the day of ET were stratified into four quartiles according to the 25th, 50th and 75th percentiles. All the categorical variables were compared with a Chi-square test between groups. The Student’s t-test was used to compare continuous variables. To analyze the impact of serum P_4_ on the day of ET defined by the four quartiles on LBR a multivariate logistic regression analysis was performed with all the potential confounding variables. The women’s age and BMI, cause of infertility, endometrial thickness, serum E_2_ and P4 quartiles, and number of MII oocytes and transferred embryos were included in the regression analysis. The receiving operating characteristic (ROC) curve was applied to define the predictive capability of serum P_4_ on LBR. The area under the curve (AUC) was calculated and the optimal threshold to predict LBR was defined according to sensitivity and specificity.

## Results

Overall, 258 eligible women with HRT-FET cycles were evaluated. The clinical pregnancy, miscarriage and live birth rates were 35.6 % (92/258), 1.5 % (4/258) and 34.1 % (88/258) respectively. Data were then categorized according to the presence (group I; *n* = 88) or absence (group II; *n* = 170) of a live birth. Age, BMI, number of mature oocytes (MII) and fertilization rate were found to be similar between groups. The mean level of P_4_ on ET day in patients without live birth (group II) was significantly higher compared to group I (37.1 ± 25.8 versus 27.3 ± 15.0, *P* < 0.001). Although, there was no statistically significant difference between the groups in terms of the mean level of E_2_ on ET day, despite the difference being close to significant (*P* = 0.07) (Table 1).

**Table 1:** The characteristics of women grouped according to presence of live birth

Variables*	With live birth (*n* = 88)	Without live birth (*n* = 170)	*P*-value
Women’ age (yr.)	32.4 ± 4.1	33.1 ± 4.1	0.18
Body mass index (kg/m^2^)	25.2 ± 2.1	25.0 ± 2.2	0.50
Cause of infertility, n (%)			0.47
PCOS	35 (39.8)	71 (41.8)	
Male factor	34 (38.6)	50 (29.4)	
Tubal factor	6 (6.8)	20 (11.8)	
Unexplained	8 (9.1)	15 (8.8)	
Mixed (both female and male factor)	5 (5.7)	14 (8.2)	
No. of MII oocytes	6.2 ± 1.9	6.0 ± 2.0	0.34
Basal serum FSH level ( IU/l)	5.9 ± 1.6	5.7 ± 1.7	0.26
Fertilization rate	0.76 ± 0.15	0.75 ± 0.14	0.62
Endometrial thickness (mm)	9.6 ± 1.4	9.5 ± 1.0	0.34
P_4_ level on ET day (ng/ml)	27.3 ± 15.0	37.1 ± 25.8	< 0.001
E_2_ level on ET day (pg/ml)	631.5 ± 350.5	722.5 ± 432.5	0.07
No. of transferred embryos	1.65 ± 0.47	1.59 ± 0. 49	0.30

*Significant level was considered at *p* < 0.05; values are presented as mean ± standard deviation or number (percentage)

The serum P_4_ values were divided into quartiles. The serum P_4_ ranges for each quartile were Q_1_: < 19 ng/ml (*n* = 64), Q_2_:19–29 ng/ml (*n* = 65), Q_3_:29–49 ng/ml (*n* = 65) and Q_4_: > 49 ng/ml (*n* = 64). Table 2 demonstrates the clinical outcome in patients according to their serum P quartile on ET day. The means of both age and BMI were similar between the four quartiles groups. CPRs were 29/64 (45.3 %), 31/65 (47.7 %), 20/65 (30.8 %) and 12/64 (18.8 %) respectively in the four quartiles group as well as LBRs were 27/64 (42.2 %), 30/65 (46.2 %), 20/65 (30.8 %) and 11/64 (17.2 %) respectively, which were found to be significantly lower in the fourth quartile (*P* = 0.002). However, there is no significant difference between the groups in terms of blighted ovum and miscarriage rates.

**Table 2:** The clinical outcome according to serum *P* values (ng/ml) on ET day

Variables*	Q_1_ (< 19 ng/ml) (*n* = 64)	Q_2_ (19–29 ng/ml) (*n* = 65)	Q_3_ (29–49 ng/ml) (*n* = 65)	Q_4_ (> 49 ng/ml) (*n* = 64)	*P*-value
Women’ age (yr.)	31.8 ± 3.7	32.8 ± 4.4	33.4 ± 4.2	33.5 ± 3.8	0.066
Body mass index (kg/m^2^)	25.5 ± 2.1	24.8 ± 2.0	25.0 ± 2.1	24.8 ± 2.1	0.17
Positive pregnancy rate/ET, n (%)	31 (48.4)	34 (52.3)	27 (41.5)	17 (26.6)	0.017
Blighted ovum rates/ET, n (%)	2 (3.1)	3 (4.6)	7 (10.8)	5 (7.8)	0.301
Clinical pregnancy rate/ET, n (%)	29 (45.3)	31 (47.7)	20 (30.8)	12 (18.8)	0.002
Miscarriage rate/ET, n (%)	2 (6.89)	1 (3.22)	0 (0)	1 (8.33)	0.590
Live birth rate/ET, n (%)	27 (42.2)	30 (46.2)	20 (30.8)	11 (17.2)	0.002

* Significant level was considered at *p* < 0.05

In similar way, the serum E_2_ values were divided into quartiles. The serum P_4_ range for each quartile were Q_1_: < 411 pg/ml (*n* = 64), Q_2_: 411–632 pg/ml (*n* = 65), Q_3_: 632–905 pg/ml (*n* = 65) and Q_4_: > 905 pg/ml (*n* = 64). Table 3 shows the clinical outcome in patients according to quartiles of the serum E_2_ on ET day. The means of women’s age and BMI were similar between the four quartiles groups. CPRs were 22/64 (34.4 %), 30/65 (46.2 %), 25/65 (38.5 %) and 15/64 (23.4 %) respectively, (*P* = 0.056); similarly, LBRs were 20/64 (31 %), 30/65 (46.2 %), 24/65 (36.9 %) and 14/64 (21.9 %) respectively, (*P* = 0.042), that were found to be significantly lower in Q_4_ group. However, there is no significant difference between groups in terms of blighted ovum and miscarriage rates.

**Table 3:** The clinical outcome according to serum E2 values (pg/ml) on ET day

Variables*	Q_1_ (< 411 pg/ml) (*n* = 64)	Q_2_ (411–632 pg/ml) (*n* = 65)	Q_3_ (632–905 pg/ml) (*n* = 65)	Q_4_ (> 905 pg/ml) (*n* = 64)	*P*-value
Women’ age (yr.)	32.7 ± 4.2	32.8 ± 4.1	32.3 ± 3.9	33.7 ± 4.0	0.29
Body mass index (kg/m^2^)	25.0 ± 2.1	25.2 ± 2.0	24.7 ± 2.0	25.0 ± 2.1	0.17
Positive pregnancy rate/ET, n (%)	29 (45.3 %)	33(50.7 %)	29 (44.6 %)	18(28.1 %)	0.056
Blighted ovum rate/ET, n (%)	7 (10.9 %)	3 (4.61 %)	4 (6.15 %)	3 (4.68 %)	0.428
Clinical pregnancy rate/ET n (%)	22 (34.4)	30 (46.2)	25 (38.5)	15 (23.4)	0.056
Miscarriage rate/ET n (%)	2 (9.09 %)	0 (0 %)	1 (4 %)	1 (6.66 %)	0.431
Live birth rate/ET n (%)	20 (31 %)	30 (46.2)	24 (36.9)	14 (21.9)	0.042

*statistically significant difference; *p* < 0.05

When all of the parameters were entered in a multivariate logistic regression model to identify which factors affect the live birth outcomes, the serum level of progesterone on ET day was the only significant variable. The patients in the highest quartile of progesterone, (Q_4_) 76 %, were less likely to have live birth compared to those in the second quartile of progesterone (Q_2_) (OR: 0.24; CI: 0.10–0.65, *P* = 0.001) (Table 4).

**Table 4:** Multivariable logistic regression analysis for detecting prognostic factors regarding live birth rate

Variables *	B	Odds ratio	(95 % confidence interval )	*P*-value
Women’ age	-0.04	0.95	(0.90–1.02)	0.16
Women’ body mass index	0.05	1.04	(0.89 − 0.12)	0.55
Cause of infertility				
Polycystic ovary syndrome		-	Reference group	-
Male factor	0.32	1.37	(0.76–2.50)	0.28
Tubal factor	-0.49	0.60	(0.22–1.65)	0.32
Unexplained factor	0.07	1.08	(0.41–2.79)	0.87
Mixed factor (both male and female)	− 0.32	0.72	(0.24–2.17)	0.56
Number of MII oocyte	0.07	1.07	(0.92–1.25)	0.34
Number of transferred embryos	0.15	1.16	(0.67-2.0)	0.57
Endometrial thickness on ET day	0.09	1.09	(0.90–1.31)	0.34
Progesterone value quartile (Q_1_) on ET day (< 19 ng/ml)	-0.16	0.851	(0.42–1.70)	0.650
Progesterone value Q_2_ on ET day (19–29 ng/ml)	-	Reference group	-	-
Progesterone value Q_3_ on ET day (29–49 ng/ml)	-0.65	0.51	(0.25–1.06)	0.073
Progesterone value Q_4_ on ET day (> 49 ng/ml)	-1.41	0.24	(0.10–0.54)	0.001
Estradiol value Q_1_ on ET day (< 411 pg/m)	-	Reference group	-	-
Estradiol value Q_2_ on ET day (411–632 pg/ml)	0.60	1.82	(0.87–3.83)	0.11
Estradiol value Q_3_ on ET day (632–905 pg/ml)	0.33	1.39	(0.65–2.96)	0.38
Estradiol value Q_4_ on ET day (> 905 pg/ml)	-0.43	0.65	(0.28–1.46)	0.30

*statistically significant difference; *p* < 0.05

**Fig. 1:**
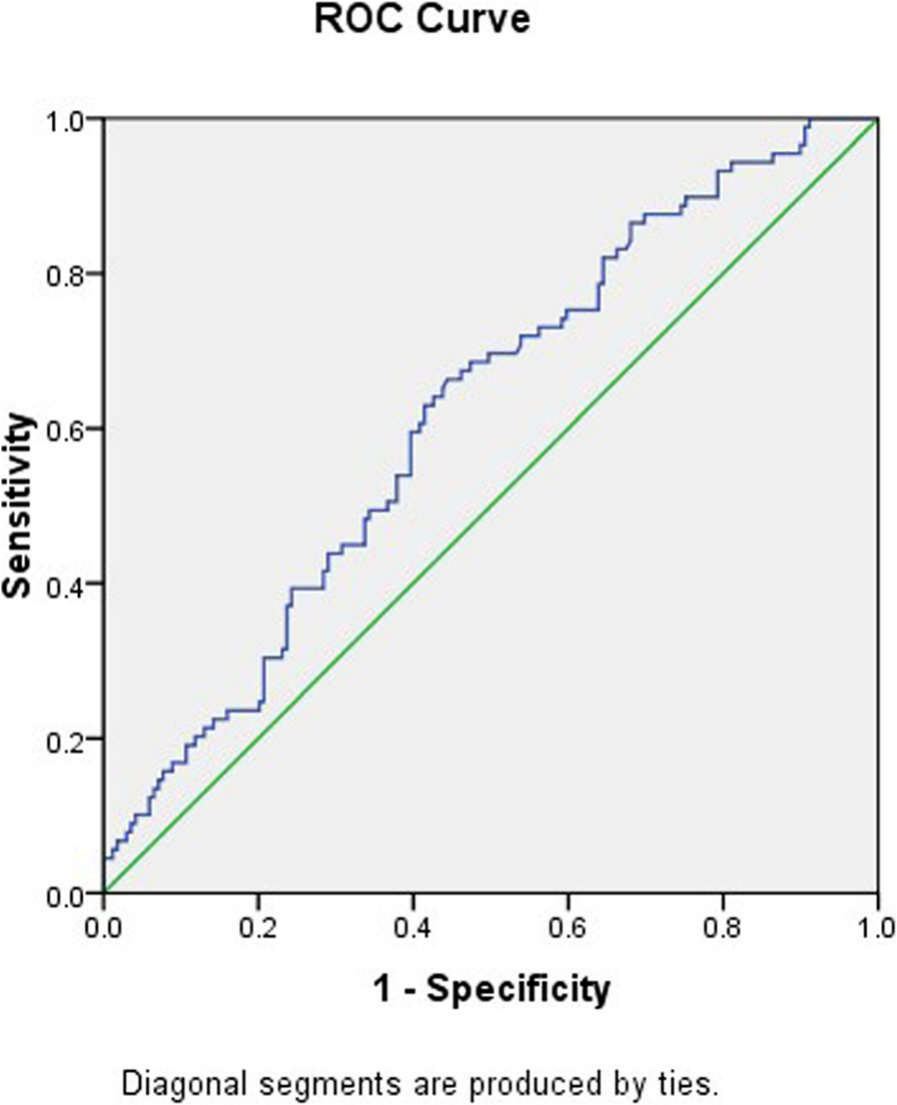
Receiver operating characteristic (ROC) curve for prediction of the live birth rate using serum progesterone levels on the day of embryo transfer. AUC = area under the curve. The serum progesterone cut-off point (32.5 ng/ml) with 70 % sensitivity and 50 % specificity was significant predictor (AUC = 0.61, 95 % CI: 0.54–0.68, *P* = 0.002)

The ROC curve showed a significant predictive value of serum P_4_ levels on the day of ET for LBR, with an AUC = 0.61 (95 % CI: 0.54–0.68, *P* = 0.002). The optimal serum P_4_ threshold, with 70 % sensitivity and 50 % specificity, for LBR was 32.5 ng/ml. The LBR around this threshold was 42.2 % versus 23.4 % for a serum P_4_ < 32.5 or ≥ 32.5 ng/ml (*P* = 0.02) (Fig. 1).

## Discussion

We conducted this study to investigate whether P_4_ and E_2_ levels measured on embryo transfer day could be correlated to pregnancy outcomes following FET cycles with the same endometrial preparation and luteal phase support. We identify a significant association between serum P_4_ levels, CPR and LBR. However, these relationships were not significant in the case of estradiol levels. In our study, patients with progesterone levels in the highest quartile (Q4) had a significantly lower likelihood of CPR and LBR compared with those in the second quartile (Q2). The current study has, additionally, shown that women with a progesterone concentration ≥ 32.5 ng/ml have significantly lower clinical pregnancy and live birth rates. It is worth noting that in our study, the level of progesterone in the lowest quartile was 19 ng/ml, and the mean of serum progesterone level was higher than in previous studies, as only 14 patients (5.4 %) had the minimum progesterone level (< 10.3 ng/ml). This could be due to the fact that a combined approach of both vaginal and IM administration of progesterone for HRT endometrial preparation was employed.

The current literature regarding the optimal range for P_4_ level on the day of ET in FET cycles using HRT regimen for endometrial preparation is limited and, indeed, controversial. The first study conducted by Kofinas et al. in a retrospective study, 213 patients were evaluated and underwent single euploid embryo frozen transfer cycles with programed endometrial preparation using 50 mg IM progesterone, and they concluded that P_4_ levels > 20 ng/ml on the day of transfer were associated with decreased ongoing pregnancy and live birth rates [5]. Similarly, Yovich et al.[3], investigated 529 FET cycles with single blastocyst embryo transfer and a unique HRT regimen with vaginal pessaries containing 10–20 mg micronized 17-beta –eosteradiol and 400 mg micronized progesterone was used for all the participants. Their analyses showed that mid-luteal serum progesterone levels below 50 nmol/l (or 15.7 ng/ml) and above 99 nmol/l (or 31.1 ng/ml) were associated with decreased implantation rates. In their study, the maximum level of progesterone (> 31.1 ng/ml) with determinant impact on implantation rate [3], was similar to the results yielded in this study. These observations suggest that either very low or very high level of progesterone concentrations can impair endometrial maturation in the implantation window. One interpretation is that low progesterone may delay or impede, while excess progesterone levels may accelerate, development of endometrium, and consequently either delay or advance the implantation window. Indeed, implying that both environments may impede synchronization of embryo and endometrial asynchronous [3]. In agreement with Yovich et al. [3], more consideration should be given when planning progesterone supplements during assisted reproductive procedures, and the clinicians may require to adjust their regimen and synchronization the day of transfer to regulate serum progesterone concentration.

Furthermore, Labarta et al. in a prospective study, evaluated 244 donor-oocyte recipients who underwent FET cycles after artificial endometrial preparation with estradiol valerate and vaginal micronized progesterone (400 mg/12 h). They found that a serum P_4_ level < 9.2 ng/ml on the day of ET was associated with reduced ongoing pregnancy rate [2]. Similarly, Cedrin-Durerin et al. assessed 277 FET cycles using micronized esteradiol (2 mg daily) and vaginal micronized progesterone (600 mg daily) for endometrial preparation, and concluded that a serum P_4_ level less than 10 ng/ml on the day of ET was associated with significantly lowered pregnancy and live birth rates [10]. In agreement with this, Gaggiotti-Marre and colleagues examined 244 FET cycles with exactly the same HRT regimen, and demonstrated that a low serum P_4_ value on the day prior to ET ≤ 10.64 ng/ml was associated with decreased pregnancy and live birth rates following frozen-thawed euploid embryo transfer. Moreover, they found that women in the lower P_4_ quartile had a significant higher miscarriage rate compared with those in the higher quartiles [7]. Recently, Boynukalin et al., in a prospective study, evaluated 168 patients who underwent a single euploid FET cycle with HRT endometrial preparation using estradiol valerate and 100 mg of IM progesterone. Their data analysis showed that patients with serum P_4_ levels < 13.6 ng/ml prior to ET had a significantly reduced likelihood of ongoing pregnancy [6].

As mentioned above, the previous studies have reported that low P level prior to FET is associated with poor pregnancy outcome. Most of the previous studies administered vaginal progesterone for endometrial preparation [2, 3, 7, 10], except for Kofinas et al.[5] and Boynukalin et al.[6] studies that used IM progesterone. Furthermore, in the present study, the endometrial preparation was performed by combining both vaginal and IM progesterone. Therefore, the differences in the methodology of the previous studies have made it difficult to compare the results. The upper limit of progesterone in fourth quartile group in this study was similar to two previous studies that used IM progesterone for endometrial preparation. It is well known that the progesterone concentration in endometrial tissue after using vaginal progesterone supplementation is significantly higher than those with IM progesterone administration, whereas; serum progesterone levels are approximately four times higher with IM progesterone compared with vaginal administration [15, 16]. Therefore, serum progesterone cut-off points will differ according to the route of progesterone administration [9]. As Shapiro and colleagues have reported, the monitoring of serum P_4_ levels is unprofitable [17]. In the case of trans-vaginal administration of drugs, despite the low serum P_4_ levels, the endometrial concentration of P_4_ were higher than the intramuscular injection cases, due to the uterine first-pass effect [18–20].

Evidence from the natural cycles in fertile women demonstrated that low levels of progesterone are necessary to achieve endometrial receptivity for embryo implantation [21]. Hull et al. in a classical study showed that a lower threshold of 9.4 ng/ml was optimal for fertility in natural cycles [22]. Regarding FET cycles, Ramezanali et al. [23] found that there was no significant difference in P_4_ levels on the day of ET between pregnant and non-pregnant women (8.7 ± 0.5 versus 8.2 ± 0.5 ng/ml) in a longitudinal study of 101 modified natural FET cycles. In artificial cycles, current evidence suggests that P_4_ levels of > 5 ng/ml provide an acceptably primed endometrium, resulting in endometrial luteinization and receptivity, which did not differ from that achieved by very higher levels [24, 25]. However, the question of whether this is functionally sufficient for implantation and maintenance of pregnancy in artificial cycles remains unanswered [2].

Contrary to some previous studies, this study found no association between progesterone quartiles groups and miscarriage rate. In previous studies, patients in the lowest quartile of progesterone had significantly higher miscarriage rates. Due to the fact that only a small number of patients in our study had minimum reported threshold of progesterone concentration (< 10 ng/ml), with most participants having favorable serum levels, the results from this study cannot fully allow for clarification of the effects of progesterone levels at the minimum threshold.

In agreement with previous findings, in this study there was a wide range of serum progesterone levels amongst participants despite administration of same dose of progesterone for all patients. The exact reason for this is uncertain, however the variability of drug absorption in patients with differing BMIs and metabolic variations might help explain this [3, 6]. As Boynukalin et al. stated, serum P_4_ levels on ET were related to BMI and women with a higher BMI would likely benefit from higher doses of progesterone upon beginning HRT. Therefore, further pharmacokinetic research is required to define optimal dosage [6]. Nevertheless, the wide range of serum progesterone values highlight that the progesterone drug uptake, distribution and metabolism can vary immensely between patients, making predicting luteal progesterone concentrations impossible without proper monitoring [9].

Interestingly, the negative impact of high serum E_2_ levels was also observed in the present study, with patients in the highest quartile (Q_4_) having the lowest LBR. However, after multivariate logistic regression analysis, the E_2_ levels on the day of ET had no significant predictive value for LBR. A review of the literature showed that the results were controversial in this area. In a retrospective study, Bocca et al., evaluated the relationship between E_2_ (late follicular phase) levels and pregnancy outcomes in HRT cycles. The results revealed that late follicular phase serum E_2_ levels did not predict pregnancy outcomes in HRT cycles [26]. In line with this, Niu et al., concluded that estradiol monitoring in FET cycle using HRT without pretreatment with gonadotropin hormone (GnRH) agonist is unnecessary [27]. In a recent retrospective cohort study, He and colleagues found that the concentration of serum E_2_ on the day of embryo transfer cannot serve as an indicator to predict the outcomes of artificial FET cycles [28]. Contrastingly, Fritz et al., reported that elevated E_2_ levels in artificial autologous FET cycles are associated with lower ongoing pregnancy and live birth rates, and suggested estradiol levels should be monitored during artificial FET cycles [29]. Since the timing of the measurements and the route of E_2_ administrations in the previous studies have varied, further prospective studies with larger sample sizes are warranted to reach a consensus in this area.

A major limitation of the current study is that the transferred embryos were not determined to be euploid. However, we have included the patients with top quality blastocyst embryos for transfer.

Additionally, we used both IM and vaginal progesterone for endometrial preparation, which precludes comparing these results with the previous studies. The clinical implications of these findings could suggest that a maximum progesterone value appears to be associated with lower live birth rate under these treatment conditions, and it can be a warning that increasing dose of progesterone should be performed with caution and careful monitoring.

In conclusion, this study demonstrates that high serum progesterone (≥ 32.5 ng/ml) on ET day in HRT–FET cycles significantly reduces the chance of live birth following blastocyst transfer. Since the timing and type of HRT regimen in previous studies have varied, the lower and upper threshold of progesterone concentrations prior to ET cannot be conclusively determined. The measurement of P_4_ levels and its monitoring during FET cycles is essential to predict the outcome of pregnancy and allow for individualized luteal phase support to be determined for each patient. However, the results are still controversial regarding the necessity of screening for serum E_2_ levels before ET, and further prospective studies are required.

## Data Availability

The datasets used and/or analyzed during the current study are available from the corresponding authors on reasonable request.

## Abbreviations

FET: Frozen embryo transfer
HRT: Hormonal replacement therapy
AC: Artificial-cycle
IM: Intramuscular
E_2_: Estradiol
P_4_: Progesterone
ET: Embryotransfer
ROC: Receiving operating characteristic
AUC: The area under the curve
LBR: Live birth rate
CPR: Clinical pregnancy rate
